# Social Isolation Impairs Oral Palatal Wound Healing in Sprague-Dawley Rats: A Role for miR-29 and miR-203 via VEGF Suppression

**DOI:** 10.1371/journal.pone.0072359

**Published:** 2013-08-09

**Authors:** Linglan Yang, Christopher G. Engeland, Bin Cheng

**Affiliations:** 1 Department of Oral Medicine, the Affiliated Hospital of Stomatology, Sun Yat-sen University, Guangzhou, Guangdong, China; 2 Guangdong Provincial Key Laboratory of Stomatology, Guangzhou, Guangdong, China; 3 Center for Wound Healing and Tissue Regeneration, College of Dentistry, University of Illinois at Chicago, Chicago, Illinois, United States of America; 4 Department of Periodontics, College of Dentistry, University of Illinois at Chicago, Chicago, Illinois, United States of America; 5 Department of Women, Child, Family Health Science, University of Illinois at Chicago, Chicago, Illinois, United States of America; Centers for Disease Control and Prevention, United States of America

## Abstract

**Objective:**

To investigate the effects of social isolation on oral mucosal healing in rats, and to determine if wound-associated genes and microRNAs (miRNAs) may contribute to this response.

**Methods:**

Rats were group housed or socially isolated for 4 weeks before a 3.5 mm wound was placed on the hard oral palate. Wound closure was assessed daily and tissues were collected for determination of gene expression levels and miRNAs (i.e., miR-29a,b,c and miR-203). The predicted target of these microRNAs (i.e., vascular endothelial growth factor A, VEGFA) was functionally validated.

**Results:**

Social isolation stress delayed the healing process of oral palatal mucosal wounds in rats. Lower mRNA levels of interleukin-1β (IL1β), macrophage inflammatory p r o t e i n-1α (MIP1α), fibroblast growth factor 7 (FGF7), and VEGFA were found in the biopsied tissues of isolated animals on days 1 and/or 3 post-wounding. Intriguingly, the isolated rats persistently exhibited higher levels of miR-29 family members and miR-203. Our results confirmed that *VEGFA* is a direct target of these miRNAs, as both miR-29a,c and miR-203 strongly and specifically suppressed endogenous VEGFA expression *in vitro*.

**Conclusions:**

This study in rats demonstrates for the first time that social isolation delays oral mucosal healing, and suggests a potential role for healing-associated gene and miRNA interactions during this process via modulation of VEGF expression.

## Introduction

Wound healing involves complex cellular and molecular interactions that are influenced not only by physical health but also by psychological state [1]. For example, restraint stress has been reported to delay dermal wound healing in mice [2]. Recently, in an elegant review, it was shown that stress moderately impairs human wound healing, with an effect size of r = 0.42 [3]. Studies suggest that a dysregulation of pro-inflammatory cytokines, chemokines, growth factors, and often a high bacterial burden, play a role in stress-impaired dermal healing [4-6].

Mucosal repair is necessary in most non-aesthetic surgical outcomes, and the vast majority (90-95%) of all infections start at mucosal surfaces [7]. So extending the findings of isolation stress to mucosal tissues is important. It is now generally recognized that mucosal wounds heal much more rapidly, with less inflammation and scarring, than skin [8-10]. While anatomical differences in dermal and mucosal repair have been described [8], the molecular basis of the healing process of mucosal wounds is less well understood. In humans, psychological stress has been shown to impair oral mucosal healing [11]. Similar results were found in individuals reporting higher depressive symptoms [12]. Despite such findings, the underlying mechanisms by which stress affects mucosal repair remain unclear.

MicroRNAs (miRNAs) are endogenous short non-coding RNAs, which play an important role in regulating normal development and physiology, as well as disease pathologies. MiRNAs have been recently shown to play pivotal roles in cutaneous wound repair [13], and aberrant expression of miRNAs may result in disorganized or poor healing. MiRNAs such as miR-16, miR-21, miR-130a [14], and miR-200b [15] have been identified as important in dermal wound healing models. However, the underlying mechanisms by which miRNAs act in the healing process remain poorly understood, especially regarding their function in mucosal tissue repair.

Perceived social isolation (i.e., loneliness) is a known risk factor for many illnesses (e.g., cardiovascular disease) and has been associated with increased morbidity and mortality in older adults [16,17]. Similarly, social isolation in rodents has been shown to reliably impair dermal wound healing [18-21, personal observations in mice - unpublished data]. To address these issues, we developed an oral palatal wound model in rats that were either group housed (non-stress) or socially isolated for four weeks (stress). Wound closure was monitored and gene expression determined in tissue post-injury for pro-inflammatory cytokines [interleukin-1β (IL1β), interleukin 6 (IL6), tumor necrosis factor-α (TNFα)], chemokines [macrophage inflammatory p r o t e i n-1α (MIP1α), monocyte chemotactic protein-1 (MCP1), CXCL1 (KC)], growth factors [fibroblast growth factor 7 (FGF7), vascular endothelial growth factor A (VEGFA)], and alpha smooth muscle actin (α-SMA) (important for wound contraction). All of these genes are important for wound healing and are highly expressed early in the repair process. To date, the effect of social isolation on mucosal repair has not been reported. In this study, we hypothesized that social isolation delays oral mucosal wound healing, and healing-associated genes and miRNAs (i.e., miR-29 and miR-203) play a role in this process.

## Materials and Methods

### Ethics statement

This study was conducted in accordance with institutional guidelines and approved by the Ethical Review Committee, Guanghua School of Stomatology, Sun Yat-sen University (Approval number ERC2012-16). All surgery was performed under chloral hydrate anesthesia, and all efforts were made to minimize suffering.

### Animals

Adult Sprague-Dawley male rats (6-8 weeks of age, 260-300 g) were obtained from the Experimental Animal Center of Sun Yat-sen University and randomly assigned to group (5/cage) or individual housing for 4 weeks prior to, and throughout this study. All rats were given free access to food and water. The facility was maintained on a 12/12-h light/dark cycle (lights on at 7 AM).

### Body Weights, Wounding, Tissue Biopsies

Rats were weighed every 3 days from the beginning of the study until sacrificed. At wounding, each rat was intraperitoneally anesthetized with 10% chloral hydrate (0.3 ml/100 g), and one full-thickness wound was placed on the oral hard palate using a sterile 3.5-mm biopsy punch (Miltex Instrument Company, York, PA, USA). On a daily basis, wounds were visually inspected under a 10× magnifier by the same investigator until considered healed. During these assessments, rats were briefly anesthetized by inhaling ether. The investigator was kept blind to the housing condition when wounds were inspected. Wounds were considered fully healed when they were completely closed, and there were no signs of erosions or ulcers. To determine intra-rater reliability for wound closure, the rater visually assessed all subjects at two different time points at day 7 and these data were evaluated using weighted Kappa statistics. The *K*w value was 0.82, suggesting there was a high intra-rater reliability.

Blood samples were obtained at 10: 00 AM before isolation (baseline), on the second day of isolation, and on days 1, 3, 5, 8 and 10 post-wounding. Rats were briefly restrained (less than 2 min) in polystyrene tubes and blood was taken from the tail vein. Approximately 80 µl of whole blood was collected from each rat and serum samples were stored at -80^°^ C for corticosterone analysis by ELISA.

In separate animals, the entire wound was harvested using a sterile 6.0-mm biopsy punch (Miltex Instrument Company) on day 1, 3, or 5 post-wounding. The excised tissue was immediately placed in 1 ml of Trizol (Invitrogen, Carlsbad, CA, USA), flash frozen in liquid nitrogen, and stored at -80^°^ C for qRT-PCR analysis. Animals were euthanized afterwards.

### Cell Culture and Transfection

HEK293 cells were maintained in DMEM/F12 supplemented with 10% FBS, 100 U/mL penicillin and 100 µg/mL streptomycin (GIBCO, Carlsbad, CA, USA) at 37° C in a humidified incubator containing 5% CO2. For functional analysis, miR-29 (a, b, c) mimics, miR-203 mimics and non-targeting miRNA mimics (Dharmacon, Lafayette, CO, USA) were transfected into cells using DharmaFECT Transfection Reagent 1 (Dharmacon) per the manufacturer’s instructions.

### ELISA

Corticosterone levels in serum were measured using ELISA kits (IB79175, IBL-America, Minneapolis, MN, USA) according to the manufacturer’s instructions. OD values were determined at 450 nm on an ELISA plate reader. Corticosterone concentrations were quantified by comparison to the standard curves. Samples were analyzed in triplicate.

### qRT-PCR

For gene expression, total RNA was extracted using the miRNeasy Mini Kit (Qiagen, Hilden, Germany) and reverse transcribed using a Transcriptor First Strand cDNA Synthesis kit (Roche, Mannheim, Germany) according to the manufacturer’s instructions. Real-time PCR was performed using a Roche 480 System (Roche) with 2 µl of 1:5 diluted cDNA, which was mixed with a TaqMan Universal PCR Master Mix, No AmpErase (Applied Biosystems, Foster City, CA, USA), primers and probe for GAPDH and the target gene, and RNase-free water to yield a 20-µl reaction volume. All of the real-time primers and probes (Table 1) were designed and produced by Bioperfectus Technologies (Jiangsu, China). The suppressor of cytokine signaling 3 (SOCS3) Taqman gene expression assay (cat. # 4331182) was purchased from Applied Biosystems. Amplification was performed in duplicate under the following conditions: 50^o^C (2 min), 95^o^C (10 min), 40 alternating cycles of 95^o^C (15 s) and 60^o^C (1 min). Relative mRNA levels of target genes were assessed by normalizing transcript Ct values to those for transcription of glyceraldehyde-3-phosphate dehydrogenase (GAPDH) (ΔCT) and comparing the results (2^-ΔCT^) directly. For miRNA expression, a quantitative 2-step RT-PCR assay using mirVanaTM qRT-PCR microRNA Detection Kit was used as per the manufacturer’s protocol (Ambion, Austin, TX, USA). Specific primer sets for miR-29a, miR-29b, miR-29c, miR-203 and U6 were obtained from Ambion. The relative expression level of miRs was determined using the 2^-ΔΔCT^ analysis method, where U6 was used as an internal reference.

**Table 1:** Primer and probe sequences used for real-time PCR.

Primer/probe		Sequence
IL1β	forward	GGATGATGACGACCTGCTAGTGT
	reverse	TGGAGAGCTTTCAGCTCACATG
	probe	CAGCTGCACTGCAGGCTTCGAG
TNFα	forward	AACTTCGGGGTGATTGGTCC
	reverse	CTGAGTGTGAGGGTCTGGGC
	probe	AGTTCCCAAATGGGCTCCCTCTCA
IL6	forward	GTTGCCTTCTTGGGACTGATG
	reverse	CTGTTGTGGGTGGTATCCTCTG
	probe	TGTTGACAGCCACTGCCTTCCC
MCP1	forward	CTCTCTTCCTCCACCACTATGC
	reverse	GTGGGGCATTAACTGCATCTG
	probe	CACGCTTCTGGGCCTGTTGTTCA
MIP1α	forward	TCCACCACTGCCCTTGCT
	reverse	CGTCCATAGGAGAAGCAGCA
	probe	TCTGCACCATGGCGCTCTGGA
KC	forward	GATTCACTTCAAGAACATCCAGAGT
	reverse	GTGGCTATGACTTCGGTTTGG
	probe	TGATGCCGCCAGGACCCCA
α-SMA	forward	AGCATCCGACCTTGCTAACG
	reverse	CATACATGGCAGGGACATTGAA
	probe	CGCCGCTGAACCCTAAGGCC
FGF7	forward	TCTATAATGCGCAAATGGATACTGA
	reverse	CGAGGTGGAAGCACGGTCT
	probe	CGGATCCTGCCGACTCCGCTC
VEGFA	forward	GGGCTGCTGCAATGATGAA
	reverse	TCCGCATGATCTGCATAGTGA
	probe	CCTGGAGTGCGTGCCCACGT
GAPDH	forward	TCCTACCCCCAATGTATCCG
	reverse	CCTTTAGTGGGCCCTCGG
	probe	CGCCTGGAGAAACCTGCCAAGTATG

### Luciferase Reporter Assay

Luciferase-expressing vectors were constructed as follows. A BglII-to-NheI fragment containing the HSV thymidine kinase (TK) promoter was inserted between BglII and HindIII sites of pGL3-Basic (Promega, Madison, WI, USA) to create pGL-TK, in which luciferase cDNA, followed by a SV40-derived polyadenylation signal, is expressed from the TK promoter. Regions covering the entire rat *VEGFA* 3’ UTR were amplified by PCR and inserted between XbaI and BamHI sites of pGL3-TK to replace the SV40 polyadenylation signal and create pGL-VEGFA. The constructs containing mutated microRNA binding sites were established by amplifying the whole vector excluding the 8-bp seed region from a pair of primers containing an additional restriction enzyme site. Thereafter, the product was digested using that restriction enzyme and ligated to recircularize the plasmid such that the seed region was replaced with the restriction enzyme site. Using lipofectamine 2000 (Invitrogen, Carlsbad, CA, USA), cells were transfected with the reporter constructs containing either the targeting sequence from the *VEGFA* 3’ UTR (named WT) or its mutant. The pRL-TK vector (Promega) was co-transfected as a control for transfection efficiency. The luciferase activities were then determined using a Lumat LB 9507 Luminometer (Berthold Technologies, Bad Wildbad, Germany).

### Western Blots

Western blots were performed as standard protocol using antibodies specific to VEGFA (Novus Biologicals, Littleton, CO, USA) and β-actin (Sigma, St. Louis, MO, USA). For semi- quantification, the western blot bands were quantified using a ChemiDoc XRS System (Bio-Rad, Laboratories, Hercules, CA) equipped with Quantity One software (version 4.6.3).

### Statistical Analysis

Statistical comparisons of differences in serum corticosterone levels were analyzed using repeated measures, treating Day (eight time points) as a within-subjects measure and Group (control, isolation) as a between-subjects measure. Analyses of mRNA levels were performed by analyte using ANOVA, treating both Day (1,3,5) and Group as between-subject measures. Post-hoc tests were performed only when significant main effects or interactions were evident that involved Group. Chi-square tests were used to assess differences in the healing rates between groups (i.e., healed vs. not healed). All hypothesis tests were 2-tailed, and the data were determined to be statistically significant when *p* < 0.05. Error bars represent the standard error of the mean (SEM). SPSS 18.0 (Chicago, IL, USA) was used for all analyses.

## Results

### Oral Palatal Wound Closure

**Figure 1:**
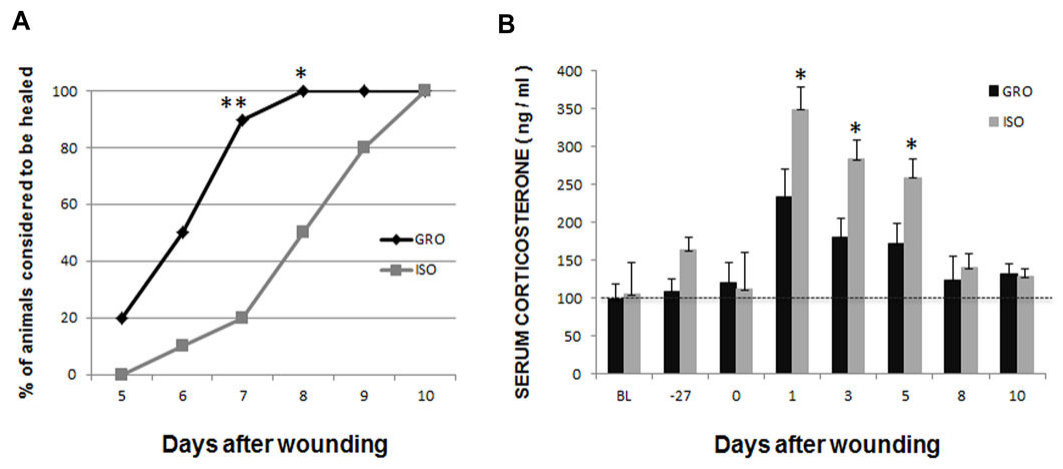
Oral palatal wound closure and serum corticosterone levels. (A) Closure of palatal wounds was significantly slower in isolated rats (ISO) than in group-housed rats (GRO). Compared with group-housed rats, a lower proportion of wounds were closed on days 7 and 8 in isolated rats (n=10/group; * *p*<0.05, ** *p*<0.01). (B) Serum corticosterone levels were significantly higher in isolated rats on days 1, 3 and 5 as compared to controls (n=5/group; * *p*<0.05). BL: baseline.

The overall rate of wound closure was markedly slower in isolated rats than in controls (*p* < 0.01). It took the isolated rats 31.2% longer than controls to be considered healed (6.4 vs. 8.4 days). As a result, on days 7 and 8 a significantly lower proportion of isolated animals were considered healed compared to non-stressed controls (Figure 1A).

### Body Weight and Serum Corticosterone Levels

There was no significant effect of isolation on body weight compared to controls over the 6-week experimental period (data not shown). When examining serum corticosterone levels, a significant Day x Group interaction was apparent [F(7,56)=3.50, p<0.01)]. Post-hoc analysis revealed that corticosterone levels were significantly higher in isolated rats as compared to controls for the first five days post-wounding (Figure 1B).

### Alteration of Healing-associated Genes in Early Healing

**Figure 2:**
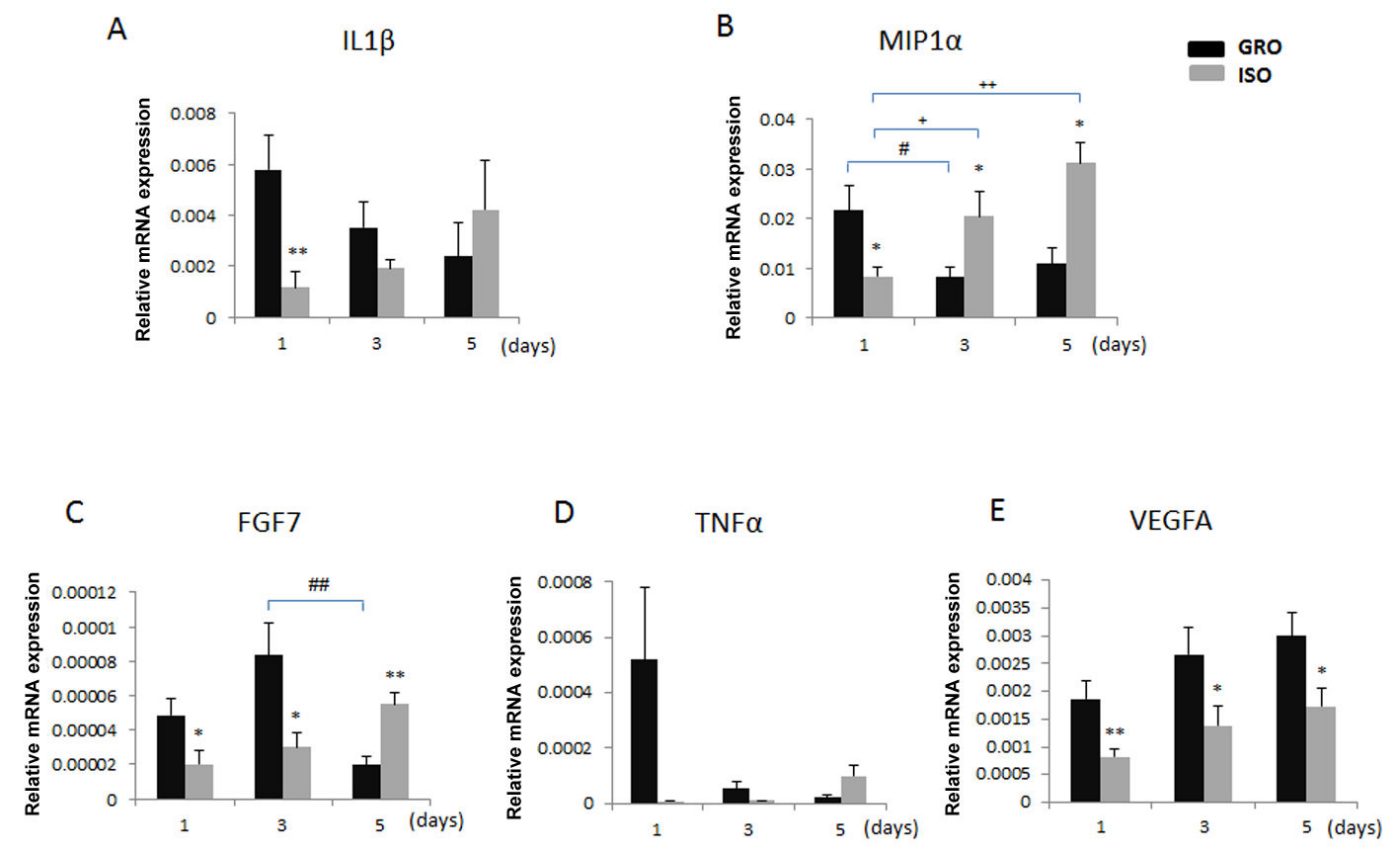
Expression levels of healing-associated genes in isolated (ISO) and group-housed (GRO) rats on Days 1, 3 and 5 post-wounding. (A) IL1β; (B) MIP1α; (C) FGF7; (D) TNFα; and (E) VEGFA. Error bars represent SEM. * indicates a significant difference between stress conditions. ^#^ and ^+^ indicate significant differences between days within group-housed and isolated rats, respectively (n=10/group; *, ^#^, ^+^, *p*<0.05; **, ^+ +^, ^##^ *p*<0.01; ^###^ *p*<0.001).

Using quantitative real-time PCR to examine mRNA levels, we found Day x Group interactions for IL1β [F(2,59)=3.36, p<0.05], TNFα [F(2,59)=4.04, p<0.05], MIP1α [F(2,59)=10.88, p<0.001], and FGF7 [F(2,59)=9.47, p<0.001]. In addition, there was a main effect of Group for VEGF [F(1,59)=16.47, p<0.001]. Post-hoc analyses revealed markedly lower mRNA level of IL1β in the wounded mucosa of isolated rats (Figure 2A) on day 1. A reduced level of MIP1α mRNA was observed on day 1 in isolated rats; conversely, MIP1α levels on days 3 and 5 were higher in isolated animals. mRNA levels of FGF7 were markedly lower in isolated rats on day 1 and day 3, and by day 5 an increased expression of FGF7 was observed in the isolated rats (Figure 2C). In particular, elevated gene expression for FGF7 and MIP1α appeared to be delayed in isolated rats compared with controls. mRNA levels for VEGFA was lower in the stress condition on all days (1 through 5) (Figure 2E). We also measured IL6, αSMA, KC and MCP1 mRNA levels in the wounded mucosa. These analytes did not differ between controls and isolated rats (not shown).

### Isolation Stress and Levels of miR-29 and miR-203

**Figure 3:**
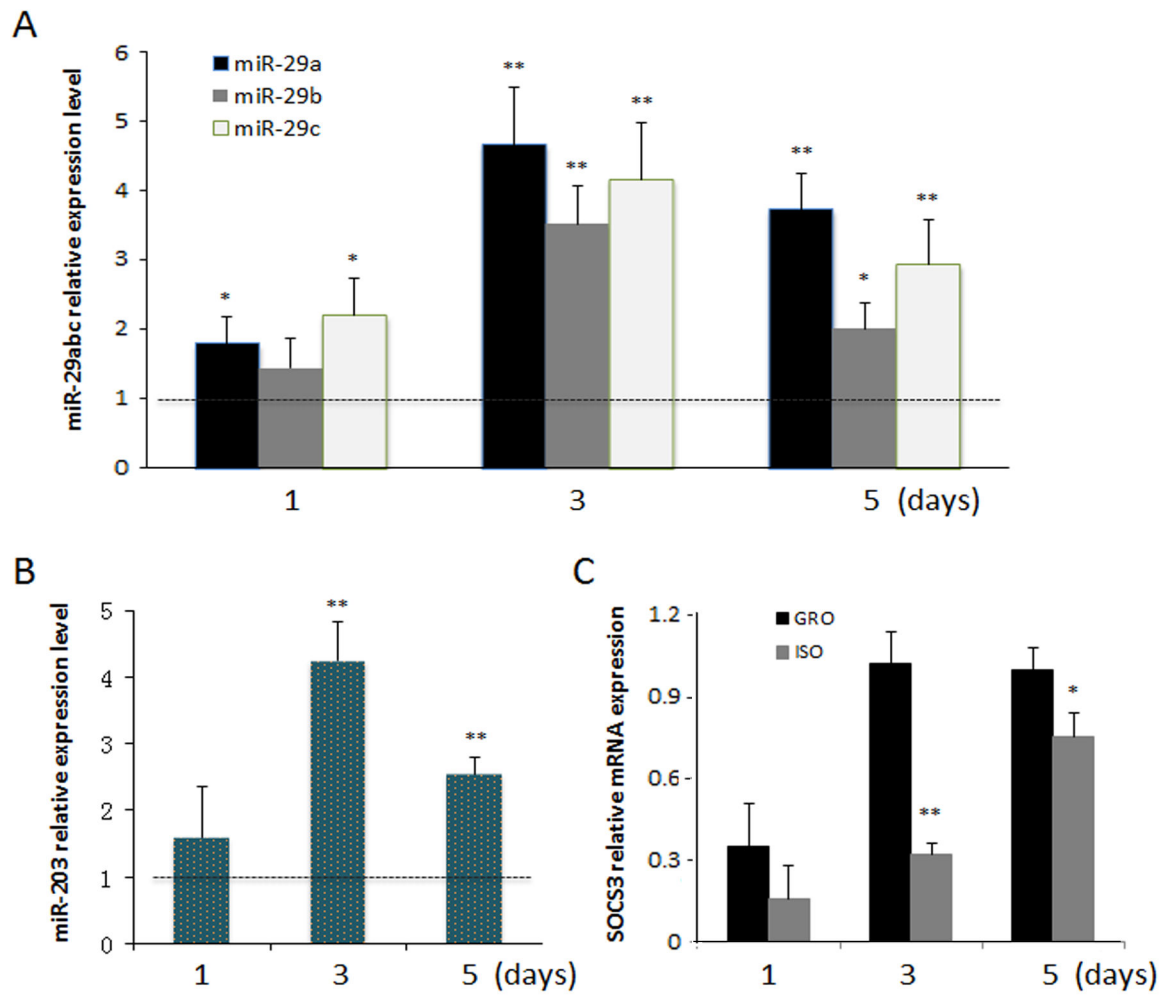
Expression of miR-29 family members, miR-203 and SOCS3 mRNA in wounded tissues by qRT-PCR. Relative expression levels of miR-29 family members (A) and miR-203 (B) in isolated (ISO) rats are in comparison to control levels expressed as 1 on the graph. U6 was used as an internal reference. (C) SOCS3 mRNA relative expression, GAPDH was used as a reference gene. (n=10/group [ISO&GRO], * indicates a significant difference between stress conditions. * *p*<0.05; ** *p*<0.01).

Compared to group housed controls, isolated rats persistently exhibited higher levels of miR-29a and miR-29c on all days in wounded mucosa (Figure 3A). Overexpression of miR-29b was detected on days 3 and 5. Expression level of miR-203 was also markedly higher on days 3 and 5 (4.2-fold and 2.6-fold, respectively) (Figure 3B). Corresponding to the higher miR-203 levels, mRNA levels of SOCS3, which is a known direct target of miR-203, were found to be lower in isolated rats on days 3 and 5 (0.32 ± 0.04 and 0.75 ± 0.09, respectively) compared to control rats.

### miR-29a,c and miR-203 Target VEGFA

**Figure 4:**
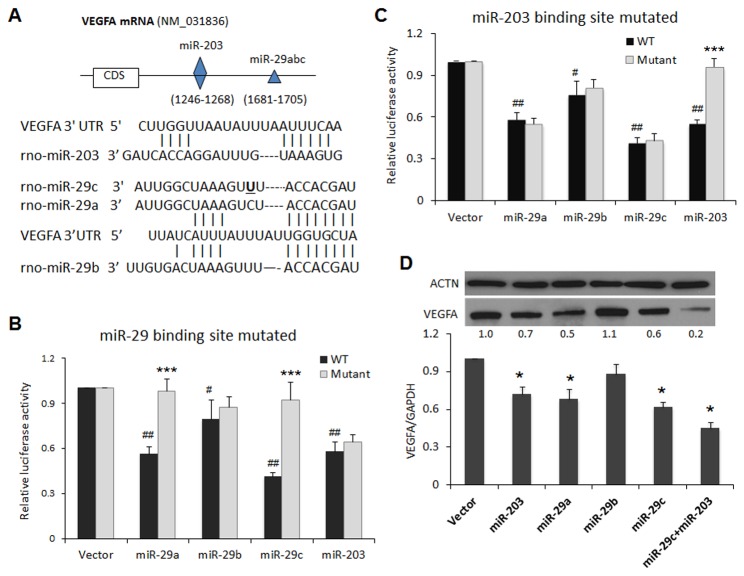
MiR-203 and miR-29 directly target VEGFA mRNA. (A) The predicted miR-203 and miR-29 targeting sequences located in the 3’-untranslated region (3’-UTR) of VEGFA mRNA. (B–C) Dual luciferase reporter assays were performed as described in *Materials and Methods* section. Cells were co-transfected with constructs containing the predicted targeting sequence (WT) or mutated targeting sequence (Mutant) cloned into the 3’-UTR of the reporter gene, along with miRNA mimics of miR-29 or miR-203. Mutation of the putative miR-203 binding site abolished the effect of miR-203 while leaving the action of all three miR-29 isoforms unaffected (B). Similarly, mutation of the putative miR-29 binding site abolished the action of the miR-29 isoforms (C). (D) Western blot analyses (upper panel) and qRT-PCR (lower) were performed to examine the effects of miRNA on VEGFA protein and gene expression in cells that were treated with miRNA mimics or control mimics. * Indicates significant differences between WT and Mutant group, ^#^ indicates significant differences between vector and each miRNA in WT cells (*, ^#^. *p* < 0.05; ***, ^##^ *p* < 0.001). Data represent at least three independent experiments with similar results.

Based on bioinformatics analysis, a highly conserved miR-29 isoform-targeting sequence and a miR-203-targeting sequence were identified in the *VEGFA* mRNA 3’ UTR (Figure 4A). To identify miRNAs capable of directly targeting these sequences, a dual-luciferase reporter assay was performed. This assay quantified the ability of overexpressed miRNAs to reduce firefly luciferase activity from an mRNA bearing the *VEGFA* 3′ UTR. The whole 3′ UTR of rat *VEGFA* was cloned downstream of the firefly luciferase coding sequence. miR-29a, miR-29c (Figure 4B) and miR-203 (Figure 4C) significantly reduced activity of firefly luciferase by directly targeting the rat *VEGFA* 3′ UTR. MiR-29c exerted the largest effect, decreasing luciferase activity by at least 60% (*p*<0.001) (Figure 4B). Both miR-29a and miR-203 reduced luciferase activity by ≥ 40% (*p*<0.001) (Figure 4B, C). To provide further confirmation of the specificity of the direct interactions between the miRNAs and *VEGFA* 3′ UTR, we mutated the predicted binding sites and repeated the luciferase assays. In both the putative miR-29 and miR-203 binding sites, the 6 to 8 nucleotides of the “seed region” were replaced with a restriction enzyme site with minimal complementarity to the miRNA sequence (see Materials and Methods). As expected, mutation of the putative miR-203 binding site abolished the effect of miR-203 while leaving the action of all three miR-29 isoforms unaffected (Figure 4B). Similarly, mutation of the putative miR-29 site led to abolishing the action of the miR-29 isoforms (Figure 4C).

### miR-29a,c and miR-203 Suppress Endogenous VEGFA

We further evaluated the ability of each of the above miRNAs to target endogenous VEGFA. The miRNA-induced change in *VEGFA* mRNA expression was confirmed by qRT-PCR. As shown in Figure 4D, ectopic transfection of miR-29a, miR-29c and miR-203 in HEK293 cells led to a significant decrease in VEGFA protein expression. Furthermore, co-transfection of miR-29c and miR-203 mimics produced an additive effect on reducing VEGFA expression *in vitro*.

## Discussion

The current study was designed to investigate the role of social isolation stress on oral mucosal wound healing. We show here that mucosal wound closure was markedly delayed in socially isolated rats compared with non-isolated controls. We also found that serum corticosterone levels were higher in isolated rats as compared to controls for the first five days post-wounding. This indicates that isolation dysregulated the stress response which occurred following wounding. University examination stress has been shown to negatively affect a similar model of wound repair in humans, delaying healing by 40% [11]. Social isolation as a stressor has been shown to impair dermal wound healing in hamsters [18], rats [19,21], and mice ([20], unpublished observations). The present results support the concept that social isolation delays wound healing (in this study by 31%), and extend these findings for the first time to mucosal tissues.

Profiling studies have described significant gene expression alterations in different dermal wound models [22-24]. A comparison of the transcriptomes of oral mucosa and skin wounds showed evidence of tissue-specific differences in the genetic response to injury [25]. Oral mucosal wounds contain less infiltrating inflammatory cells and exhibit lower levels of pro-inflammatory cytokines (e.g., IL1β, TNFα) and chemokines (e.g., MIP1α) than skin [8,22]. As expected, we found that isolated rats exhibited a reduced early increase in the expression of these genes, and thus a delay in peak expression. In addition, decreased VEGFA mRNA levels and a delayed increase in gene expression for FGF7 were evident in isolated rats. Similar mechanistic delays in dermal wound healing have been reported in restraint-stressed mice (2,6).

Compared to skin, oral mucosal wounds are characterized by an altered expression of healing-associated genes, and a distinct healing pattern [26,27]. For instance, mucosal wounds heal faster than dermal wounds, with less inflammation and little or no scarring [8,28]. These variations between tissues potentially reflect a different expression of specific miRNAs. To test this, we selected and determined the levels of two miRNAs in wounds: miR-29 and miR-203. MiR-29 is a typical multifunctional miRNA which is involved in regulating the epithelial-mesenchymal transition, cellular differentiation, extracellular matrix remodeling, and angiogenesis [29,30]. Reduced expression of miR-29 family members was recently reported in different fibrotic organs [31-33]. MiR-203 is the first identified skin- and keratinocyte-specific miRNA. This miRNA is upregulated in inflammatory and immune-mediated diseases [34-38]. Intriguingly, both miR-29 and miR-203 are important regulators of wound-specific cell functions and the cytokine network [38,39]. In accordance with these findings, our results demonstrate that miR-29 family members and miR-203 were persistently overexpressed across healing in isolated rats. This suggests there are novel roles of miR-29 and miR-203 in isolation-impaired healing. It should be pointed out that we could not exclude other VEGF-targeted miRNAs from playing a role in the delayed healing process, as we did not perform miRNA microarray analysis to screen the up-regulated miRNAs between isolated and control rats.

Interestingly, in this study, we identified a negative correlation between levels of VEGFA and the expression of miR-29a,c and miR-203 in wounds. VEGFA is a pivotal mediator of inflammation and angiogenesis, processes intimately involved in tissue repair. Recent studies indicate that VEGFA is a target of proteases that are present in the microenvironment of human chronic non-healing wounds [40]. In this study, VEGFA was the only healing-associated mRNA targeted by miR-29 and miR-203 (out of the nine that were tested). In addition, the conserved sites of VEGFA were predicted by multiple miRNA target prediction algorithms (e.g., Targetscan, miRanda, RNA22, etc), and these miRNA-mRNA target pairs have potential high binding ability and thermal stability. Our in vitro experiments confirmed that these miRNAs suppress the expression of endogenous VEGFA. Such suppression was most apparent in isolated rats, as lower expression of VEGFA was evident in these animals from days 1 to 5 post-injury. Although there are presumably additional targets regulated by miR-29 and miR-203, our experiments suggest that VEGFA suppression is mediated, at least in part, by overexpression of these miRNAs. Of note, these miRNAs also influence the production of other inflammatory molecules such as SOCS3. SOCS3 is a negative regulator of the STAT3 signaling pathway, which has critical functions in the regulation of inflammation, cell growth, and differentiation [41]. Suppression of SOCS3 by miR-203 results in increased and prolonged skin inflammatory responses [39]. Consistent with these reports, the current results in isolated rats showed that gene expression for SOCS3 was suppressed while miR-203 was upregulated on days 3 and 5. Thus, as reported here in conditions of isolation stress, miR-203 overexpression may have contributed to the delay and eventual prolongation of inflammation, and reduced expression of angiogenic factors, via its actions on VEGFA. The role for miR-203 and SOCS3 delaying healing need be further investigated.

In conclusion, this study demonstrates that social isolation impairs oral mucosal wound healing. The unique expression pattern of gene-miRNA interactions in injured tissues represents a novel aspect of the complex regulatory network involved in mucosal repair. These data suggest that overexpression of miR-29 and miR-203 contributes to isolation-induced delays of wound healing, partially by suppressing VEGFA. Considering the important role of miR-29 and miR-203 in both dermal and mucosal repair, these results suggest a putative mechanism for isolation-induced healing impairments through the modulation of VEGFA. Inhibition of the miRNAs identified in this study may provide a target for future therapeutics that are directed at speeding the repair of mucosal tissues. This could be particularly useful for conditions of inflamed oral tissue repair such as periodontitis.

## References

[1] Koschwanez HE, Broadbent E (2011) The use of wound healing assessment methods in psychological studies: A review and recommendations. Br J Health Psychol 16: 1-32. doi:10.1348/135910710X524633. PubMed: 21226781. 21226781 10.1348/135910710X524633

[2] Padgett DA, Marucha PT, Sheridan JF (1998) Restraint stress slows cutaneous wound healing in mice. Brain Behav Immun 112: 64-73. PubMed: 9570862. 10.1006/brbi.1997.0512 9570862

[3] Walburn J, Vedhara K, Hankins M, Rixon L, Weinman J (2009) Psychological stress and wound healing in humans: A systematic review and meta-analysis. J Psychosom Res 67: 253-271. doi:10.1016/j.jpsychores.2009.04.002. PubMed: 19686881. 19686881 10.1016/j.jpsychores.2009.04.002

[4] Kiecolt-Glaser JK, Loving TJ, Stowell JR, Malarkey WB, Lemeshow S et al. (2005) Hostile marital interactions, proinflammatory cytokine production, and wound healing. Arch Gen Psychiatry 62: 1377-1384. doi:10.1001/archpsyc.62.12.1377. PubMed: 16330726. 16330726 10.1001/archpsyc.62.12.1377

[5] Peng C, He Q, Luo C (2011) Lack of keratinocyte growth factor retards angiogenesis in cutaneous wounds. J Int Med Res 39: 416-423. doi:10.1177/147323001103900209. PubMed: 21672345. 21672345 10.1177/147323001103900209

[6] Rojas IG, Padgett DA, Sheridan JF, Marucha PT (2002) Stress-induced susceptibility to bacterial infection during cutaneous wound healing. Brain Behav Immun 16: 74-84. doi:10.1006/brbi.2000.0619. PubMed: 11846442. 11846442 10.1006/brbi.2000.0619

[7] Bosch JA, de Geus EJ, Veerman EC, Hoogstraten J, Nieuw Amerongen AV (2003) Innate secretory immunity in response to laboratory stressors that evoke distinct patterns of cardiac autonomic activity. Psychosom Med 65: 245-258. doi:10.1097/01.PSY.0000058376.50240.2D. PubMed: 12651992. 12651992 10.1097/01.psy.0000058376.50240.2d

[8] Szpaderska AM, Zuckerman JD, DiPietro LA (2003) Differential injury responses in oral mucosal and cutaneous wounds. J Dent Res 82: 621-626. doi:10.1177/154405910308200810. PubMed: 12885847. 12885847 10.1177/154405910308200810

[9] Mak K, Manji A, Gallant-Behm C, Wiebe C, Hart DA et al. (2009) Scarless healing of oral mucosa is characterized by faster resolution of inflammation and control of myofibroblast action compared to skin wounds in the red Duroc pig model. J Dermatol Sci 56: 168-180. doi:10.1016/j.jdermsci.2009.09.005. PubMed: 19854029. 19854029 10.1016/j.jdermsci.2009.09.005

[10] Wong JW, Gallant-Behm C, Wiebe C, Mak K, Hart DA et al. (2009) Wound healing in oral mucosa results in reduced scar formation as compared with skin: evidence from the red Duroc pig model and humans. Wound Repair Regen 17: 717-729. doi:10.1111/j.1524-475X.2009.00531.x. PubMed: 19769724. 19769724 10.1111/j.1524-475X.2009.00531.x

[11] Marucha PT, Kiecolt-Glaser JK, Favagehi M (1998) Mucosal wound healing is impaired by examination stress. Psychosom Med 60: 362-365. PubMed: 9625226. 9625226 10.1097/00006842-199805000-00025

[12] Bosch JA, Engeland CG, Cacioppo JT, Marucha PT (2007) Depressive symptoms predict mucosal wound healing. Psychosom Med 69: 597-605. doi:10.1097/PSY.0b013e318148c682. PubMed: 17766687. 17766687 10.1097/PSY.0b013e318148c682

[13] Banerjee J, Chan YC, Sen CK (2011) MicroRNAs in skin and wound healing. Physiol Genomics. 43(10): 543-556. doi:10.1152/physiolgenomics.00157.2010. PubMed: 20959495. 20959495 10.1152/physiolgenomics.00157.2010 PMC3110888

[14] Pastar I, Khan AA, Stojadinovic O, Lebrun EA, Medina MC et al. (2012) Induction of Specific MicroRNAs Inhibits Cutaneous Wound Healing. J Biol Chem 287: 29324-29335. doi:10.1074/jbc.M112.382135. PubMed: 22773832. 22773832 10.1074/jbc.M112.382135 PMC3436197

[15] Chan YC, Roy S, Khanna S, Sen CK (2012) Downregulation of endothelial microRNA-200b supports cutaneous wound angiogenesis by desilencing GATA binding protein 2 and vascular endothelial growth factor receptor 2. Arterioscler Thromb Vasc Biol 32: 1372-1382. doi:10.1161/ATVBAHA.112.248583. PubMed: 22499991. 22499991 10.1161/ATVBAHA.112.248583 PMC3399424

[16] Grippo AJ, Carter CS, McNeal N, Chandler DL, Larocca MA et al. (2011) 24-hour autonomic dysfunction and depressive behaviors in an animal model of social isolation: implications for the study of depression and cardiovascular disease. Psychosom Med 73: 59-66. doi:10.1097/PSY.0b013e31820019e4. PubMed: 21097661. 21097661 10.1097/PSY.0b013e31820019e4 PMC3088487

[17] Cacioppo JT, Hawkley LC, Norman GJ, Berntson GG (2011) Social isolation. Ann N Y Acad Sci 1231: 17-22. doi:10.1111/j.1749-6632.2011.06028.x. PubMed: 21651565. 21651565 10.1111/j.1749-6632.2011.06028.x PMC3166409

[18] Detillion CE, Craft TK, Glasper ER, Prendergast BJ, DeVries AC (2001) Social facilitation of wound healing. Psychoneuroendocrinology 29: 1004-1011. PubMed: 15219651. 10.1016/j.psyneuen.2003.10.003 15219651

[19] Vitalo A, Fricchione J, Casali M, Berdichevsky Y, Hoge EA et al. (2009) Nest making and oxytocin comparably promote wound healing in isolation reared rats. PLOS ONE 4: e5523. doi:10.1371/journal.pone.0005523. PubMed: 19436750. 19436750 10.1371/journal.pone.0005523 PMC2677672

[20] Glasper ER, Devries AC (2005) Social structure influences effects of pair-housing on wound healing. Brain Behav Immun 19(1): 61-68. doi:10.1016/j.bbi.2004.03.002. PubMed: 15581739. 15581739 10.1016/j.bbi.2004.03.002

[21] Levine JB, Leeder AD, Parekkadan B, Berdichevsky Y, Rauch SL et al. (2008) Isolation rearing impairs wound healing and is associated with increased locomotion and decreased immediate early gene expression in the medial prefrontal cortex of juvenile rats. Neuroscience 151: 589-603. doi:10.1016/j.neuroscience.2007.10.014. PubMed: 18063315. 18063315 10.1016/j.neuroscience.2007.10.014 PMC3923447

[22] Chen W, Fu X, Ge S, Sun T, Zhou G et al. (2007) Profiling of genes differentially expressed in a rat of early and late gestational ages with high-density oligonucleotide DNA array. Wound Repair Regen 15: 147-155. doi:10.1111/j.1524-475X.2006.00195.x. PubMed: 17244330. 17244330 10.1111/j.1524-475X.2006.00195.x

[23] Roy S, Khanna S, Rink C, Biswas S, Sen CK (2008) Characterization of the acute temporal changes in excisional murine cutaneous wound inflammation by screening of the wound-edge transcriptome. Physiol Genomics 34: 162-184. doi:10.1152/physiolgenomics.00045.2008. PubMed: 18460641. 18460641 10.1152/physiolgenomics.00045.2008 PMC2494843

[24] Roy S, Patel D, Khanna S, Gordillo GM, Biswas S et al. (2007) Transcriptome-wide analysis of blood vessels laser captured from human skin and chronic wound-edge tissue. Proc Natl Acad Sci U S A 104: 14472-14477. doi:10.1073/pnas.0706793104. PubMed: 17728400. 17728400 10.1073/pnas.0706793104 PMC1964861

[25] Chen L, Arbieva ZH, Guo S, Marucha PT, Mustoe TA et al. (2010) Positional differences in the wound transcriptome of skin and oral mucosa. BMC Genomics 11: 471-477. doi:10.1186/1471-2164-11-471. PubMed: 20704739. 20704739 10.1186/1471-2164-11-471 PMC3091667

[26] Chen L, Gajendrareddy PK, Dipietro LA (2012) Differential expression of HIF-1α in skin and mucosal wounds. J Dent Res 91: 871-876. doi:10.1177/0022034512454435. PubMed: 22821237. 22821237 10.1177/0022034512454435 PMC3420394

[27] Szpaderska AM, Walsh CG, Steinberg MJ, Dipietro LA (2005) Distinct patterns of angiogenesis in oral and skin wounds. J Dent Res 84: 309-314. doi:10.1177/154405910508400403. PubMed: 15790734. 15790734 10.1177/154405910508400403

[28] Schrementi ME, Ferreira AM, Zender C, DiPietro LA (2008) Site-specific production of TGF-beta in oral mucosal and cutaneous wounds. Wound Repair Regen 16: 80-86. doi:10.1111/j.1524-475X.2007.00320.x. PubMed: 18086295. 18086295 10.1111/j.1524-475X.2007.00320.x

[29] Kriegel AJ, Liu Y, Fang Y, Ding X, Liang M (2012) The miR-29 family: genomics, cell biology, and relevance to renal and cardiovascular injury. Physiol Genomics 44: 237-244. doi:10.1152/physiolgenomics.00141.2011. PubMed: 22214600. 22214600 10.1152/physiolgenomics.00141.2011 PMC3289120

[30] Kogure T, Costinean S, Yan I, Braconi C, Croce C et al. (2012) Hepatic miR-29ab1 expression modulates chronic hepatic injury. J Cell Mol Med 16: 2647-2654. doi:10.1111/j.1582-4934.2012.01578.x. PubMed: 22469499. 22469499 10.1111/j.1582-4934.2012.01578.x PMC3923513

[31] Wang B, Komers R, Carew R, Winbanks CE, Xu B et al. (2012) Suppression of microRNA-29 expression by TGF-β1 promotes collagen expression and renal fibrosis. J Am Soc Nephrol 23: 252-265. doi:10.1681/ASN.2011010055. PubMed: 22095944. 22095944 10.1681/ASN.2011010055 PMC3269175

[32] Van Rooij E, Sutherland LB, Thatcher JE, DiMaio JM, Naseem RH et al. (2008) Dysregulation of microRNAs after myocardial infarction reveals a role of miR-29 in cardiac fibrosis. Proc Natl Acad Sci U S A 105: 13027-13032. doi:10.1073/pnas.0805038105. PubMed: 18723672. 18723672 10.1073/pnas.0805038105 PMC2529064

[33] Roderburg C, Urban GW, Bettermann K, Vucur M, Zimmermann H et al. (2011) MicroRNA profiling reveals a role for miR-29 in human and murine liver fibrosis. Hepatology 53: 209-218. doi:10.1002/hep.23922. PubMed: 20890893. 20890893 10.1002/hep.23922

[34] Yi R, Poy MN, Stoffel M, Fuchs E (2008) A skin microRNA promotes differentiation by repressing 'stemness'. Nature 452: 225-229. doi:10.1038/nature06642. PubMed: 18311128. 18311128 10.1038/nature06642 PMC4346711

[35] Primo MN, Bak RO, Schibler B, Mikkelsen JG (2012) Regulation of pro-inflammatory cytokines TNFα and IL24 by microRNA-203 in primary keratinocytes. Cytokine 60: 741-748. doi:10.1016/j.cyto.2012.07.031. PubMed: 22917968. 22917968 10.1016/j.cyto.2012.07.031

[36] Stanczyk J, Ospelt C, Karouzakis E, Filer A, Raza K et al. (2011) Altered expression of microRNA-203 in rheumatoid arthritis synovial fibroblasts and its role in fibroblast activation. Arthritis Rheum 63: 373-381. doi:10.1002/art.30115. PubMed: 21279994. 21279994 10.1002/art.30115 PMC3116142

[37] Viticchiè G, Lena AM, Cianfarani F, Odorisio T, Annicchiarico-Petruzzelli M et al. (2012) MicroRNA-203 contributes to skin re-epithelialization. Cell Death Dis 3: e435. doi:10.1038/cddis.2012.174. PubMed: 23190607. 23190607 10.1038/cddis.2012.174 PMC3542609

[38] Sonkoly E, Ståhle M, Pivarcsi A (2008) MicroRNAs and immunity: novel players in the regulation of normal immune function and inflammation. Semin Cancer Biol 18: 131-140. doi:10.1016/j.semcancer.2008.01.005. PubMed: 18291670. 18291670 10.1016/j.semcancer.2008.01.005

[39] Sonkoly E, Wei T, Janson PC, Sääf A, Lundeberg L et al. (2007) MicroRNAs: novel regulators involved in the pathogenesis of psoriasis? PLOS ONE 2: e610. doi:10.1371/journal.pone.0000610. PubMed: 17622355. 17622355 10.1371/journal.pone.0000610 PMC1905940

[40] Eming SA, Krieg T (2006) Molecular mechanisms of VEGF-A action during tissue repair. J Investig Dermatol Symp Proc 11: 79-86. doi:10.1038/sj.jidsymp.5650016. PubMed: 17069014. 10.1038/sj.jidsymp.5650016 17069014

[41] Kubo M, Hanada T, Yoshimura A (2003) Suppressors of cytokine signaling and immunity. Nat Immunol 4: 1169-1176. doi:10.1038/ni1012. PubMed: 14639467. 14639467 10.1038/ni1012

